# Low ficolin-3 levels in early follow-up serum samples are associated with the severity and unfavorable outcome of acute ischemic stroke

**DOI:** 10.1186/1742-2094-8-185

**Published:** 2011-12-29

**Authors:** George Füst, Lea Munthe-Fog, Zsolt Illes, Gábor Széplaki, Tihamér Molnar, Gabriella Pusch, Kristóf Hirschberg, Robert Szegedi, Zoltán Széplaki, Zoltán Prohászka, Mikkel-Ole Skjoedt, Peter Garred

**Affiliations:** 1 3rd Department of Internal Medicine, Semmelwies University, Budapest, Hungary; 2 Laboratory of Molecular Medicine, Department of Clinical Immunology-7631, Rigshospitalet, University of Copenhagen, Copenhagen, Denmark; 3 Division of Clinical and Experimental Neuroimmunology, Department of Neurology, University of Pecs, Pecs, Hungary; 4 Institute of Anaesthesia and Intensive Therapy, Faculty of Medicine, University of Pecs, Pecs, Hungary; 5 Heart Center, Semmelweis University, Budapest, Hungary; 6 Department of Neurology, Kútvölgyi Clinical Centre, Semmelweis University, Budapest, Hungary; 7 Experimental Laboratory of Cardiac Surgery, University of Heidelberg, Germany

**Keywords:** stroke, ischemic stroke, outcome, complement, lectin pathway, ficolins, ficolin-2, ficolin-3, CRP

## Abstract

**Background:**

A number of data indicate that the lectin pathway of complement activation contributes to the pathophysiology of ischemic stroke. The lectin pathway may be triggered by the binding of mannose-binding lectin (MBL), ficolin-2 or ficolin-3 to different ligands. Although several papers demonstrated the significance of MBL in ischemic stroke, the role of ficolins has not been examined.

**Methods:**

Sera were obtained within 12 hours after the onset of ischemic stroke (admission samples) and 3-4 days later (follow-up samples) from 65 patients. The control group comprised 100 healthy individuals and 135 patients with significant carotid stenosis (patient controls). The concentrations of ficolin-2 and ficolin-3, initiator molecules of the lectin complement pathway, were measured by ELISA methods. Concentration of C-reactive protein (CRP) was also determined by a particle-enhanced immunturbidimetric assay.

**Results:**

Concentrations of both ficolin-2 and ficolin-3 were significantly (p < 0.001) decreased in both the admission and in the follow-up samples of patients with definite ischemic stroke as compared to healthy subjects. Concentrations of ficolin-2 and ficolin-3 were even higher in patient controls than in healthy subjects, indicating that the decreased levels in sera during the acute phase of stroke are related to the acute ischemic event. Ficolin-3 levels in the follow-up samples inversely correlated with the severity of stroke indicated by NIH scale on admission. In follow-up samples an inverse correlation was observed between ficolin-3 levels and concentration of S100β, an indicator of the size of cerebral infarct. Patients with low ficolin-3 levels and high CRP levels in the follow up samples had a significantly worse outcome (adjusted ORs 5.6 and 3.9, respectively) as measured by the modified Rankin scale compared to patients with higher ficolin-3 and lower CRP concentrations. High CRP concentrations were similarly predictive for worse outcome, and the effects of low ficolin-3 and high CRP were independent.

**Conclusions:**

Our findings indicate that ficolin-mediated lectin pathways of complement activation contribute to the pathogenesis of ischemic stroke and may be additive to complement-independent inflammatory processes.

## Background

Neuroinflammation is a key element in the ischemic cascade after cerebral ischemia that results in cell damage and death in the subacute phase.[1]

Complement activation is one of the pathological mechanisms that contribute to the ischemic/reperfusion injury in ischemic stroke [2-4]. Among other neuroinflammatory processes, the complement system is also activated during tissue injury and has recently been considered as a new potential therapeutic target in ischemic stroke [5] and in intracerebral haemorrhage [6]. Both animal experiments and observations made in stroke patients indicate that activation of the complement system is one of the mechanisms contributing to the extension of the cerebral infarct after ischemic stroke [7]. Several studies have demonstrated the essential role of complement activation in brain damage following cerebral ischemia. Such evidence includes (i) an increased expression of complement proteins and complement receptors after permanent middle cerebral artery occlusion (MCAO) [8-11] (ii) different pathological events in complement-deficient/-sufficient animals after the onset of cerebral ischemia compared to wild-type littermates: complement deficient animals are at least partially protected after transient MCAO [12-15]. (iii) In rodent experimental models, complement depletion induced using the cobra venom factor (CVF) [16,17], as well as complement inhibition by a plasma-derived C1-inhibitor [18,19], a recombinant C1 inhibitor [20], CR2-Crry [13] and intravenous immunoglobulin administration [14] were proven to exert beneficial, neuroprotective effects, indicating the protective role of complement antagonism and inhibition.

Only a few studies have explored complement activation in patients with ischemic stroke [21,22]. Recently, we found that sC5b-9 levels determined at admission exhibited a significant positive correlation with the clinical severity of stroke, as well as with the extent of the neurological deficit as determined by different scales [3]. Our findings suggested that the lectin pathway is primarily responsible for the activation of complement in ischemic stroke. In agreement with these findings, Cervera et al. [4] demonstrated both in mice and stroke patients that genetically determined MBL-deficiency is associated with a better outcome after acute ischemic stroke. In a high number of patients with ischemic stroke, Osthoff et al. [23] found that a deficiency of the mannose-binding lectin is associated with smaller infarction size and a more favorable outcome. More recently, the group of De Simoni [24] reported on the formation of functional MBL/MASP-2 complexes in plasma in mice after MCAO, and demonstrated that molecules, which strongly bound to MBL, induced significant reduction in neurological deficits and infarct volume, when administered 6 h after transient MCAO. These data support the notion that the lectin pathway plays a crucial role in the development of ischemic stroke.

Apart from MBL, the ficolins also serve as recognition molecules in the lectin complement pathway. Three different ficolins have been described in humans. Ficolin-1, -2, and -3 are derived from the genes *FCN1*, *FCN2*, and *FCN3*, respectively. In healthy individuals, ficolin-2 and -3 are present in the serum and plasma in relatively high concentrations, while the concentration of ficolin-1 is much lower [25]. Similar to MBL, the ficolins are associated with a set of three serine proteases, termed MBL-associated serine proteases (MASPs), enabling activation of the complement system. The primary activator of the lectin pathway appears to be MASP-2.

As described above, there are abundant data about the significance of MBL in ischemic stroke. The role of the ficolins, initiator molecules of the lectin complement pathway, however, has never been studied in this disease. Therefore, we measured the levels of ficolin-2 and ficolin-3 in sera from 65 patients with ischemic stroke and from controls. In order to assess the clinical significance of the results, serum concentrations of these proteins were correlated to an indirect measure of the stroke severity (NIHss), S100β concentration on day 3, which is an indicator of the size of cerebral infarct, [26,27] as well as the outcome of the disease expressed by the modified Rankin scale.

Besides complement activation, other inflammatory processes are also known to contribute to the pathogenesis of the ischemic stroke [1]. Among them, CRP-associated processes were mostly studied. In 2005, Di Napoli et al [28] summarized evidence for CRP as an independent predictor of cerebrovascular events in at-risk individuals and its usefulness in evaluating prognosis after stroke. It was also demonstrated that C-reactive protein predicts the prognosis of patients with functional disability after the first occurrence of ischemic stroke [29] and correlates to the infarct volume [30]. Recently, Ormstad et al. [31] provided evidence that CRP plays an important role in the progression of cerebral tissue injury. In addition, in our previous study [3] we found that complement activation and elevated CRP levels were independently associated with the clinical severity and different outcome measures of ischemic stroke, indicating their additive effect. Therefore, serum concentrations of CRP and its relationship to the ficolin levels were also examined here.

## Methods

### Patients and control subjects

*Patients with ischemic stroke *included in the present work were admitted to two centers: the Department of Neurology, University of **Pecs**, Hungary (39 patients:20 men and 19 women, aged 49-84 years) and the Department of Neurology, Kútvölgyi Clinical Centre, Semmelweis University, **Budapest**, Hungary (26 patients:10 men and 16 women, aged 58-87 years) **(**Table 1). The management of ischemic stroke was in accordance with the guidelines of the Stroke Council of the American Heart Association/American Stroke Association [32] None of the patients were treated by intravenous thrombolysis. Patients with stroke were enrolled upon the first occurrence of acute ischemic stroke only; all patients had neuroimaging (most of them brain MRI, but at least cranial CT). No patients had hemorrhagic infarction. All patients with definite acute clinical symptoms were enrolled regardless of etiology i.e. lacunar or territorial infarct caused by thrombosis or emboli. Exclusion criteria were infectious diseases, fever < 4 weeks before stroke, an elevated WBC, erythrocyte sedimentation rate (ESR), high-sensitivity CRP (hsCRP, cut-off value < 10 mg/L), procalcitonin on admission (cut-off value < 0.05 ng/mL), positive chest X-ray, hemorrhagic stroke defined by an acute cranial CT scan, and those who declined to participate in the study. Almost all patients had hypertension and elevated cholesterol/triglyceride levels. All patients were therefore treated for such risk factors; nevertheless the effect of such treatments on the ficolin pathway is unlikely. An evidence-based guideline [33] was followed to detect post-stroke infectious complications (in short, physical and laboratory measures including WBC, ESR, hsCRP, PCT, fever, abnormal urine, chest X-ray or positive cultures). Such complications occurred on the 4^th ^day as an average, and were located to the respiratory system and urinary tract even in the absence of catheterization; in addition, thrombophlebitis occurred in a single case.

**Table 1:** Main characteristics of the cohorts tested

Cohort	Patients with ischemic stroke	Healthy controls	Patients with severe athero-sclerosis (patient controls)
Number of subjects	65	100	134

Sex, males/females	20/19	47/53	88/46

Age, years, mean ± S.D.	69.8 ± 9.8	35.5 ± 9	69.8 ± 9.9

Median time between the onset of symptoms and blood sampling, in hours	7.0 or 8.5*		

Infection, yes/no	9/56		

Lethal outcome yes/no	7/58		

NIH scale at admission, ≥ 16 vs < 16	58/7		

Serum S100β levels, pg/ml, median (IQ range)	0,27 (0.12-0.93)		

Outcome: modified Rankin scale at discharge: 0/1/2/3/4/5/6	4/13/14/7/9/7/1		

*in the Pécs and Budapest groups, respectively.

At the time of admission, severity of stroke was assessed using the National Institutes of Health Stroke Scale (NIHSS) [34]. Blood samples were obtained at the time of admission (admission samples: the median time from the onset of symptoms was 7 hours in the Budapest cohort and 8.5 hours in the Pecs cohort), and 72 to 96 hours later (follow-up samples). Five and four patients in the Pecs and Budapest cohorts, respectively, developed infections. The outcome of disease was assessed with the modified Rankin scale [35].

Serum samples were also taken from 100 *healthy volunteers *as controls (Table 1). Additionally, 134 *patients with significant carotid atherosclerosis *served as controls (Table 1). In agreement with international guidelines, significant carotid atherosclerosis was defined as 70-100% stenosis of the carotid artery determined by Duplex scan sonography. The examination was indicated in the case of other vascular disorders or risk factors of vascular disorders. None of the patients had definite residual signs and no symptoms suggesting acute ischemia. Some of these patients had either peripheral arterial disease or coronary disease, and in these patients carotid Duplex scans were performed to detect asymptomatic severe carotid stenosis (as a common comorbidity). Some of the patients had non-specific symptoms (i.e. dizziness) or transient ischemic attack previously; in these patients diagnostic carotid Duplex scans were performed. Lacunar strokes defined by neuroimaging were no exclusion criteria.

Serum samples of the patients and of the controls were stored at -80°C in the Hungarian laboratories until transported on dry ice to Copenhagen.

The study was approved by the local ethics committees, and all patients and control subjects gave informed consent.

### Laboratory methods

The serum concentrations of the proteins ficolin-2 [36] and ficolin-3 [37] were determined by ELISA-based methods at the Laboratory of Molecular Medicine, Department of Clinical Immunology, Rigshospitalet, Copenhagen, Denmark. Briefly, microtiter plates were coated with either monoclonal anti-ficolin-2 antibody (FCN216) or monoclonal anti-ficolin-3 antibody (FCN334) in phosphate buffered saline (PBS) overnight at 4°C. Samples diluted 1:50 or 1:640 in sample buffer (PBS-T with 1% mouse serum and bovine serum) were added in triplets to washed wells and incubated for 3 hours at 37°C. Ficolin-2 was detected with biotinylated monoclonal anti-ficolin-2 antibody (FCN219) and ficolin-3 was detected with biotinylated monoclonal anti-ficolin-3 antibody (FCN334) by incubation overnight at 4°C. Washed wells were incubated for 1 hour at 37°C with HRP-conjugated streptavidin. Plates were developed for 15 min with OPD (o-phenylenediamine) substrate solution and stopped by adding 1M H_2_SO_4_. The optical density was measured at 490 nm. A standard dilution series of pooled human serum were added to each assay as were a sample control. The lower limit of detection in these assays is 5 ng/ml of ficolin-2 and 1 ng/ml of ficolin-3. The inter-assay coefficient of variation (CV) is 7.1% and 4.7% and the intra-assay CV 4.3% and 3.9% for the ficolin-2 and ficolin-3 assay, respectively.

Human S100β concentrations were measured by an ELISA method (BioVendor, Modrice, Czech Republic). In our previous study, we found that the concentration of S100β was the highest 72 hours after the onset of stroke, therefore concentration was determined at this timepoint [27].

Serum CRP concentrations were measured by particle-enhanced immunturbidimetric assay, using an automated laboratory analyzer (Roche Cobas Integra 400, Basel, Switzerland).

### Statistical evaluation of the results

Statistical analysis was performed using the GraphPad Prism 3.0 (GraphPad Software Inc, San Diego, CA, http://www.graphpad.com) and SPSS 13.0 (SPSS Inc., Chicago, IL) software. Between-group differences were evaluated by the Mann-Whitney test. Correlations between the variables were expressed using non-parametric Spearman's correlation coefficients. The categorical variables were compared with the χ^2 ^test for trend. The association between the serum concentration of selected proteins and the outcome of stroke was calculated by multiple logistic regression, adjusted for the sex and the age of the patients. All tests were two-tailed. All data are presented as median values with the 25^th ^to 75^th ^percentiles in parentheses unless stated otherwise.

## Results

### The concentrations of the proteins of the lectin pathway and CRP in the sera of patients with ischemic stroke, as compared to healthy controls and patient controls

**Figure 1:**
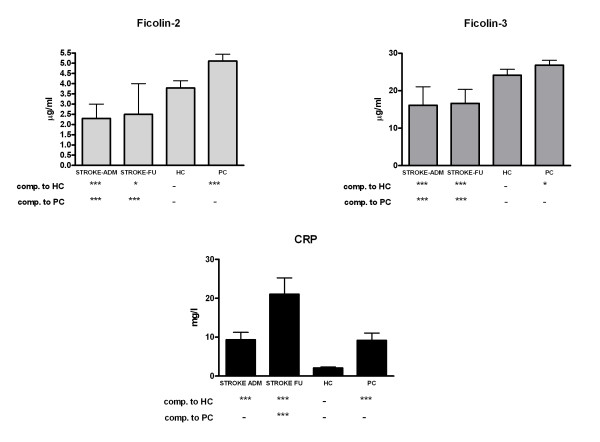
**Concentrations of ficolin-2, ficolin-3 and C-reactive protein in the sera of patients with acute ischemic stroke**. Concentrations at the time of hospital admission and on day 3, as compared to healthy controls (HC) and patient controls (PC, patients with > 70% stenosis of the carotid artery without acute stroke) are shown. P values (* < 0.05, *** < 0.01) for the non-parametric Kruskal-Wallis test followed by the Dunn post hoc test are indicated.

The serum levels of ficolin-2, ficolin-3 and CRP were measured in the samples obtained from 65 stroke patients on admission and 3-4 days later (follow-up samples), as well as in the sera of 100 healthy volunteers and 134 patient controls (patients with severe carotid atherosclerosis without acute stroke) (Figure 1). Compared to both healthy controls and patient controls, both ficolin-2 ficolin-3 levels were significantly lower both in the admission and follow-up sera of stroke patients. When all patients were considered, CRP levels were significantly higher in the admission samples than in the sera of healthy controls but were nearly equal to that measured in the sera of patient controls. By contrast in the follow up samples, CRP levels were significantly higher as compared to both control groups. When patients who developed infections were not considered, the difference between stroke patients and controls became non-significant (data not shown). As for the two controls groups, all the three variables had significantly higher concentration in the sera of patient controls than in the healthy controls, although the difference in the ficolin-3 levels was small.

### Follow-up ficolin-3 levels correlated with the indirect measures of stroke severity and infarct size

**Figure 2:**
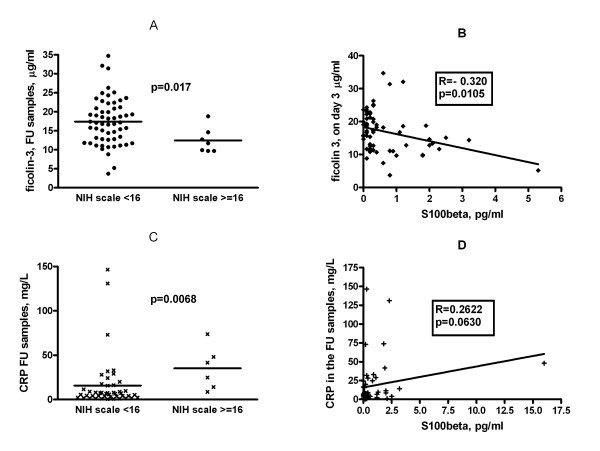
**Correlation between ficolin-3 levels with severity and outcome of stroke and size of infarct**. Panel A: Negative correlation between serum ficolin-3 levels in follow-up (FU) samples and the severity of stroke as assessed by the NIH stroke scale at admission in 65 patients with ischemic stroke. Patients with unfavorable (≥ 16) vs. favorable (< 16) NIH scale were compared. P value of Mann-Whitney test is indicated. Panel B: Negative correlation between serum ficolin-3 levels in follow-up samples and the size of cerebral infarct as assessed by the S100β level in follow-up samples. Spearman's correlation coefficient and its significance is indicated. Panel C: Positive correlation between serum CRP levels in follow-up samples and the severity of stroke as assessed by the NIH scale at admission in 65 patients with ischemic stroke. Patients with unfavorable (≥ 16) vs. favorable (< 16) NIH scale were compared. P value of Mann-Whitney test is indicated. Panel D: No significant correlation between serum CRP levels in follow-up samples and the size of cerebral infarct as assessed by the S100β level in follow-up samples. Spearman's correlation coefficient and its significance is indicated.

Ficolin-3 concentrations measured in the follow-up samples but not in the admission samples exhibited a significant, negative correlation with indirect measures of stroke severity i.e. the NIH score determined on admission (Figure 2, **panel A**). Patients were divided into two groups in a similar manner to Foerch 2005 [26]; those with a NIH scale of < 16 with relatively good expected outcome and those with NIH scale of ≥ 16 with poor expected outcome, and the ficolin-3 levels were compared accordingly. There were significantly (p = 0.017) lower ficolin-3 levels in the former than in the latter group. By contrast, no significant differences in the ficolin-2 levels (p = 0.309) were found between the two groups (data not shown).

In addition, we found significant negative correlation between ficolin-3 concentrations and the S100β level measured in the follow-up samples but not in the admission samples (Figure 2, **panel B**). The levels of ficolin-2 did not correlate with the S10B concentrations (data not shown).

CRP concentrations in follow-up samples were significantly higher in patients with high (≥ 16) NIH score (Figure 2, **Panel C**), but did not significantly correlate with the S100β levels (Figure 2, **panel D)**.

### Ficolin-3 and CRP levels in follow-up samples correlate with the outcome of acute ischemic stroke

**Figure 3:**
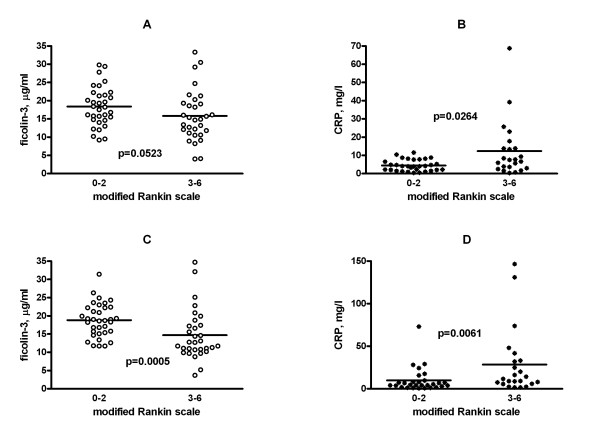
**Relationship of serum ficolin-3 and CRP levels with outcome**. Differences in ficolin 3 (left panels, A, C) and CRP (right panels, B, D) levels measured in admission samples (upper panels, A, B) and in follow-up samples (lower panel, C, D) comparing patients with a favorable (modified Rankin scale: 1 or 2) and an unfavorable (modified Rankin scale 3 to 6) outcome. In the case of the CRP calculations, nine patients with infectious complications were excluded from the analysis. The significance of the Mann-Whitney test is indicated.

The levels of the ficolins and CRP were related to the outcome of the disease, as assessed by the modified Rankin scale (Figure 3). When patients were divided according to unfavorable (3 to 6) and favorable (0 to 2) modified Rankin scores, ficolin-3 levels were lower in the former group, supporting the association with an unfavorable outcome. The difference was significant only in the follow-up samples, while almost significant in the admission samples (Figure 3, **panels A and B**). When the 9 patients, who developed infectious complications were excluded, CRP levels both in admission and follow up samples were significantly higher in the patient group with unfavorable compared to favorable outcome (Figure 3, **panels C and D**).

We confirmed these data by performing a multiple logistic regression analysis. Unfavorable (modified Rankin scale 3 to 6) vs. favorable (modified Rankin scale 0 to 2) outcome was regarded as a dependent variable, whereas ficolin-3 levels, CRP levels, age and sex were considered as independent variables (Table 2). Since both ficolin-3 and CRP levels were included in the analysis, those 9 patients who developed infectious complications were excluded.

**Table 2:** Relationship between the ficolin-3 and CRP levels and unfavorable (modified Rankin scale 3 to 6) vs. favorable (modified Rankin scale: 1 to 2) outcome of ischemic stroke as calculated by multiple logistic regression analysis

	OR (95% CI) (p value)**	
	**Admission samples**	**Follow-up samples**

ficolin 3, μg/ml	0.989 (0.776-1.020) (p = 0.093)	0.736 (0.603-0.899) (p = 0.003)

CRP, mg/L	1.226 (1.040-1.446) (p = 0.015)	1.375 (1.095-1.727) (p = 0.006)

Sex (females/males)	0.951 (0.238-3.794) (p = 0.943)	0.969 (0.207-4.535) P = 0.968)

Age, years	1.005 (0.925-1.092) (p = 0.904)	0.981 (0.902-1.066) (p = 0.652)

Multiple logistic regression analysis adjusted for sex and age was used. Nine patients with infectious complications were excluded from the analysis.

Both ficolin-3 and CRP levels measured in the follow up samples were significantly associated with the outcome of the disease: lower ficolin-3 and higher CRP values were found in the unfavorable compared to the favorable outcome group. Similar but only, marginally significant (ficolin-3) or weakly significant (CRP) associations were found when the admission samples were analyzed.

Next, in order to assess the strength of association between the low ficolin-3 and high CRP levels on the one hand and the unfavorable outcome of the disease on the other hand, we repeated the analysis as above in the follow-up samples by including ficolin-3 and CRP levels as low/high values. Ficolin-3 levels below or equal to the median (16 μg/ml for both the admission and follow up samples) were considered low, while those above the median value were considered high. CRP levels above median (7.7 mg/L) were considered high (Table 3). In the analysis, adjusted for sex and age of the patients, both the low ficolin-3 and the high CRP levels significantly predicted an unfavorable outcome, with odds ratios of 5.6 and 3.9, respectively.

**Table 3:** Relationship between the low ficolin-3 and high CRP levels and outcome of ischemic stroke

	OR*** (95% CI)	P value
Low vs. high ficolin 3*	5.628 (1.497-21.153)	0.044

High vs. low CRP**	3.949 (1.036-15.055)	0.011

Sex (females/males)	1.171 (0.306-4.491)	0.818

Age, years	1.041 (0.966-1.122)	0.294

*low ficolin 3 defined as < 16 μg/ml (median), **high CRP levels defined as > 7,7 mg/L (median), ***unfavorable (modified Rankin scale: 3 to 6) vs. favorable (modified Rankin scale: 0 to 2) outcome. Multiple logistic regression analysis adjusted to sex and age was used.

Nine patients with infectious complications were excluded from the analysis.

### Correlation between the baseline NIH score, serum S100β concentration in the follow up samples as well as the outcome of the disease

**Figure 4:**
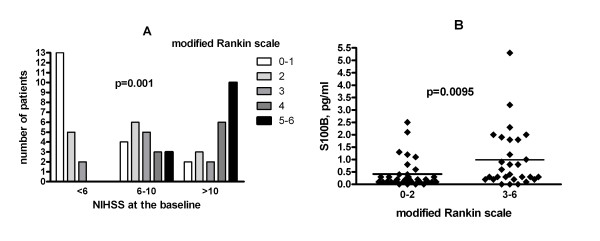
**Relationship between the baseline NIH score scale values and the 3-day serum S100β concentration with the outcome of the disease in 65 patients with ischemic stroke**. Panel A: Distribution of the patients with different outcome of the disease among patients with low (< 6), medium (6-10) and high (> 10) baseline NIHSS scale. P value for χ^2 ^test is indicated. Panel B: Differences in the S100β concentration between patients with favorable (modified Rankin score: 0-2) and unfavorable modified Rankin score: 3-6) outcome.

Finally, we assessed the relationship between the baseline NIH scale as an indirect measure of the severity of the stroke, the concentration of the S100β in the follow up samples as an indicator of the infarct size, and the outcome of the disease assessed by the modified Rankin scale (Figure 4). Both measures exhibited highly significant correlation to the outcome: patients with high baseline NIHSS scale had much worse outcome than those with low NIH scale, and patients with unfavorable outcome had higher serum S100β concentrations at 72 hours than those with a favorable outcome.

## Discussion

We report here on three novel observations: (i) the decrease of serum concentrations of two proteins of the lectin pathway during the acute phase of ischemic stroke; (ii) an inverse correlation of ficolin-3 levels obtained 3-4 days post-admission with the severity and outcome of acute ischemic stroke; (iii) the independent effect of low ficolin-3 and high CRP levels on the outcome of the disease.

As compared to healthy subjects, the serum concentrations of both ficolin-2 and ficolin-3, initiator proteins of the lectin complement pathway, were significantly lower in the samples taken from patients with ischemic stroke immediately after admission (i.e. within hours after the onset of the symptoms). The levels of these proteins did not further change during the initial 3-4 days of stroke. The differences observed between stroke patients and healthy individuals seem to be valid, since the ficolin-2 and ficolin-3 concentrations measured in the sera of healthy subjects are similar to previously reported data [38]. In addition, ficolin-2 and ficolin-3 levels were significantly lower in sera of patients with definite stroke compared to patients with severe carotid atherosclerosis without clinical event as well. The main age of this group was equal to that of stroke patients. This control group of patients exhibited even higher ficolin levels than healthy subjects. These data may suggest that the decreased levels of ficolins in the acute phase of stroke were not related to the chronic and severe atherosclerosis, but rather a decrease in ficolin-2 and ficolin-3 concentrations may happen in the very early phase of the acute ischemic event. In addition, these data indicated that the difference in ficolin concentrations comparing healthy controls and stroke patients were not related to the difference between their ages.,.

The decreased concentration of ficolins could be observed in the very early phase of ischemic stroke and remained unchanged during the next 3-4 days. It seems reasonable to surmise that this decrease was due to consumption through the binding of the molecules to the apoptotic and necrotic cells in the penumbra of the cerebral infarct [2]. Moreover, ficolin-2 and ficolin-3 have also been shown to be involved in the sequestration of dying host cells [39]. The observations made by Wang et al. [40] are of particular interest, since these authors reported that maternal plasma concentrations of ficolin-3 and ficolin-2 were significantly (p < 0.001) lower in preeclamptic pregnancies than in uncomplicated pregnancies, due to the sequestration of the proteins in placenta. Additionally, they found that both ficolins but particularly ficolin-3 were associated with ischemic placenta tissue.

According to our second observation, lower level of ficolin-3 in the follow-up samples were associated with greater size of the cerebral infarct indicated by higher S100β levels in the sera. Astrocyte-derived S100β concentration is a marker of the degree and the severity of cellular injury in acute ischemic stroke [41]. The examination of S100β protein has been accepted as a good biomarker of the infarct size [26,42-44]. The concentration of S100β is known to be the highest 72 hours after the onset of stroke [27].

In addition, ficolin-3 levels inversely correlated with the indirect measure of the severity of ischemic stroke, i.e. with the NIHSS neurological deficit score. Higher NIHSS scores define more severe deficits [34].

Additionally, a strong negative correlation was found between ficolin-3 concentration and the outcome of the disease measured with modified Rankin scale. This negative correlation indicates that low ficolin-3 levels are associated with an unfavorable prognosis. This association is most probably secondary to the negative correlation between ficolin-3 on the one hand and the severity of ischemic stroke and the infarct size on the other hand, as discussed above. It is well known that both the high baseline NIHSS score and the high serum S100β levels predict poor prognosis of ischemic stroke, which was also found in the present study (Figure 4). The lack of clinical correlates of ficolin-2 could be explained by the observation that ficolin-3 has the highest concentration and the greatest complement-activating capacity among the lectin pathway initiators [45].

Complement activation is one of the pathological mechanisms contributing to ischemic/reperfusion injury in ischemic stroke [2-4]. The selective ability for complement activation after the binding of ficolin-3 to dying cells may be responsible for the selective clinical correlation with the levels of this protein. Further studies, including simultaneous measurement of ficolin-3 levels and of the generation of complement activation products, are necessary to confirm this assumption.

Our present findings also support previous data [3,4,23], which showed that the lectin complement pathway indeed plays an important role in the pathogenesis of acute ischemic stroke; here we show that a ficolin-3-dependent activation of the lectin pathway also contributes to the pathological processes besides the previously suggested MBL-dependent activation.

Third, in accordance with the previous data [46-48] and earlier work from our groups [3,27], we measured higher CRP levels in the sera of patients obtained at admission as compared to healthy controls, and high CRP levels measured on day 3 were strongly associated with an unfavorable outcome of ischemic stroke. This latter observation is in accordance with the recent findings of Song et al. [29]. According to our present findings, the clinical associations with the low ficolin-3 and high CRP levels measured in the follow up samples are independent, indicating that they reflect two different pathways of inflammation contributing to the pathogenesis of the disease. These findings may have important therapeutic implications. Anti-inflammatory drugs have already been used for the treatment of ischemic stroke with limited success. Since many pharmacological agents, which are able to inhibit pathological complement activation are either approved for therapeutic purposes (such as C1-inhibitor [49] or eculizimab [50]) or are under clinical trials [51], these may be more efficiently used for treatment of ischemic stroke either alone or in combination with anti-inflammatory drugs.

The paper has some limitations. First of all, the number of patients tested is rather low and no late follow-up samples were collected for ficolin measurements. Nevertheless our observations are novel and may initiate a number of studies.

## Conclusions

Our findings indicate that two seemingly different but only partially identified pathways of neuroinflammation, the ficolin-3-dependent activation of lectin pathway of complement and CRP-dependent processes independently contribute to the pathogenesis and poor outcome of acute ischemic stroke. These findings may lead to the introduction of novel treatment approaches for a disease with a rather limited therapeutic arsenal at present.

## List of abbreviations

CR1: complement receptor type 1; CR2: complement receptor type 2; CRP: C-reactive protein, CVF: cobra venom factor; MCAO: middle cerebral artery occlusion; MASP: MBL-associated serine protease; MBL: mannose-binding lectin; NIHSS: National Institutes of Health Stroke Scale; OR: odds ratio.

## Competing interests

The authors declare that they have no competing interests.

## Authors' contributions

GF, ZsI and PG conceived of the study, and participated in its design and coordination, and helped to draft the manuscript; L-MT and M-OS carried out the immunoassays; GSZ, T, GP, KH, RSZ and ZSz participated in the collection and analysis of clinical data; ZP participated at the design of the study and drafting the manuscript. All authors read and approved the final manuscript.
